# Caregivers’ voices: From the world of autism spectrum disorder

**DOI:** 10.4102/curationis.v47i1.2519

**Published:** 2024-07-23

**Authors:** Patience M.M. Dira, Rorisang J. Machailo, Suegnet Scholtz

**Affiliations:** 1 School of Nursing Science, Faculty of Health Sciences, North-West University, Potchefstroom, South Africa

**Keywords:** autism, autism spectrum disorder (ASD), caregivers, children, coping mechanisms, experiences

## Abstract

**Background:**

Caring for a child with autism spectrum disorder (ASD) is a challenging and stressful task, especially in countries with limited resources. Additional research is necessary, considering the increasing prevalence of children with ASD, to gain increased knowledge of the complex difficulties faced by caregivers of ASD children and to offer insights into the coping strategies and support networks that parents utilise.

**Objectives:**

The objective of this study was to explore and describe the experiences and coping mechanisms of caregivers of children with ASD in Dr Kenneth Kaunda district, North West province, South Africa.

**Method:**

Qualitative explorative, contextual and descriptive design with purposive sampling technique and semi-structured interviews were conducted. Data were analysed following the six steps of reflexive thematic analysis.

**Results:**

Two themes were identified: Caregivers’ experiences in raising a child with autism, and caregivers’ coping in raising a child with autism.

**Conclusion:**

The research established caregivers’ experiences and coping mechanisms in raising a child with ASD and the effects on different aspects of their lives including emotional, social and financial aspects, which contribute negatively to their holistic well-being. These impediments warrant the establishment of emotional support groups, empowerment of caregivers and awareness-raising through campaigns to educate the family and the community on the diverse challenges.

**Contribution:**

The findings of this study contribute to a deeper understanding of the multifaceted challenges faced by caregivers of children with ASD and provide insights into the support systems and coping mechanisms employed by these caregivers within the socio-ecological context.

## Introduction

Autism spectrum disorder (ASD) is defined by Alli, Abdoola and Mupawose (2015:81) as developmental disability that causes impairment in a person’s social interaction and communication and manifests as controlled, standardised stereotyped patterns of behaviour. There is considerable evidence indicating an increase in reported incidences of ASD globally, as diagnostic and screening techniques become more advanced (Gobrial 2018:133). However, the unclear reports of ASD in African countries cause challenges in estimating the actual numbers of children diagnosed with ASD in both low- and middle-income countries (Shilubane & Mazibuko 2020:2). In their study, Pillay, Duncan and De Vries (2021:1076) stated that there is limited information available regarding the prevalence of ASD in South Africa. It is estimated that 2% of South Africans are affected by the disorder (Reddy, Fewster & Gurayah 2019:43). Increasingly, many families are caring for children diagnosed with ASD and may require support in doing so (Burrell, Ives & Unwin 2017:1135). Despite ASD being a significant disorder confronting many families in South Africa, there are no reliable statistics regarding its prevalence and extent in North West province. Literature highlights that raising a child with autism may be an unpleasant experience, which could result in diminished competence, increased stress, and difficulties on both the mental and physical health of caregivers (Aguiar & Pondé 2019:43). Additionally, Aderinto, Olatunji and Idowu (2023:4) mention that families and caregivers in Africa are severely burdened emotionally and financially by ASD, which increases stress levels and restricts access to support resources. Furthermore, Schlebusch, Dada and Samuels (2017:2) indicated that South African families raising children with ASD face challenges in accessing diagnostic interventions and educational services.

According to Dillenburger (2015:245), the exact cause of ASD is unknown, but it is thought to be a combination of genetic and environmental risk factors. Symptoms include deficits in social-emotional reciprocity, such as failure to sustain normal back and forth conversation, non-verbal communicative behaviours, as well as deficits in developing, maintaining and understanding relationships (Lord et al. 2018:509). The indefinite manifestation of ASD necessitates continuous dedication to providing care, including coping with stressors as well as implementing lifestyle changes to manage the child (Reddy et al. 2019:43). Caring for a child with ASD may generate a negative impact on the family’s functioning as well as the members’ emotional health and social interactions because of the behavioural problems inherent in the condition (Aguiar & Pondé 2019:43).

The authors derived this study from Bronfenbrenner’s 1992 conceptual framework of the socio-ecological model (Golden et al. 2015:9S). Social ecology models are visual representations of the constant interactions that exist between individuals, groups and environments (Golden et al. 2015:9S). Individuals are thought to influence and be influenced by the people and organisations with whom they engage, as well as the resources and institutions accessible to them and social norms and standards (Golden et al. 2015:9S). The framework is classified into five levels: individual, interpersonal, community, institutional and policy or environment level. Individual level consists of caregiver’s characteristics such as age, gender, educational level, economic resources, expectations and values, which determine caregiver response to experiences encountered and coping mechanisms utilised. According to the study of Salas et al. (2017:59), mothers of children with ASD show a significantly higher level of distress, anxiety and depression compared to fathers and as parents age, the use of cognitive restructuring techniques and social assistance decreases. In terms of economic resource, families with lower financial status tend to adopt emotional-focused coping strategies, a lack social support, and experience a lower quality of life compared to those with ordinary economic conditions (Wang, Liu & Zhang 2022:3). Higher levels of parental education were found to be linked to increased involvement and responsibility in the child’s care (Sharabi & Marom-Golan 2018:56). According to this study, caregiver is defined as a biological parent, foster parent, grandparent, step-parent or any other person responsible for caring for a child with ASD, who stays with the child for more than 6 weeks, maintains the child’s basic needs and provides ongoing full-time support to the child. Children with ASD are reported to have higher levels of behavioural and emotional difficulties than typically developing children (Hastings et al. 2020:4). These behavioural challenges generate an increase in depression and a decrease in the morale of their mothers (Yoder 2022:12). According to Bluth et al. (2013:199), researchers presumed that the presence of a child with ASD in the family was associated with elevated parental stress. The statement, despite its accuracy in numerous cases, is insufficient as it excludes additional strains not related to the presence of a child with ASD (Bluth et al. 2013:199).

It is for this reason that the authors of this article sought to explore and describe the experiences and coping mechanisms of caregivers of children with ASD in Dr. Kenneth Kaunda district in the North West province, South Africa.

## Research methods and design

A qualitative exploratory, descriptive and contextual design was used to explore and describe the experiences and perceptions on challenges linked to ASD, particularly where there is little published research about the disorder (Doyle et al. 2020:444). The method facilitates an exact understanding of the experiences and coping mechanisms of caregivers of children with ASD, which is a principal focus in this study.

### Setting

The study was conducted at a public hospital located in North West province, in Dr. Kenneth Kaunda district. Tertiary care institutions are furnished with personnel and facilities for advanced investigation and treatment, enabling the provision of specialised consultative care, typically through referrals from primary or secondary medical care institutions (Swain & Kar 2018:251).

There are various healthcare services that are provided by the hospital, such as speech therapy, occupational therapy, psychology and paediatrics departments in which caregivers of children with autism bring the children for these services. The research was conducted within the facility as it is the North West province’s referral hospital.

### Population and sampling

The study comprised ten caregivers of children diagnosed with ASD between the ages of 3 years and 17 years and either a male or a female child. Caregivers were of 18 years and above and brought their children at the public hospital located in Dr. Kenneth Kaunda, in North West province. Caregivers were able to understand English or any other African language and willing to be audio-recorded. Caregivers of typically developing children, caregivers of children having physical disabilities and other neurological developmental disorders were excluded. A purposive sampling technique was utilised as participants were selected based on a preconceived idea that they could answer the research questions (Chukwuere, Sehularo & Manyedi 2022:2).

### Data collection

Individual face-to-face semi-structured exploratory interviews were conducted by the first author in a safe and secure environment that allowed caregivers of children with ASD to respond to questions freely. The interviews were recorded as consented by participants and lasted ± 45–60 min, in which five open-ended questions were asked according to the predetermined interview guide. The central questions asked were: ‘What are your experiences as a caregiver of a child with autism spectrum disorder?’ and ‘How do you cope personally, emotionally, socially and financially as a caregiver of a child diagnosed with autism spectrum disorder?’ Interviews were conducted in English and South Sotho, languages that participants were comfortable in expressing their experiences. At the conclusion of the interview, the first author asked the participants if they had any more information to contribute or any questions to ask. Interviews were conducted until data saturation was reached. Saturation denotes the absence of new data (Saunders et al. 2018:1895).

Data collection started in August 2022 and was completed in the first week of December 2022. Recruitment took place within the healthcare facility by means of recruitment posters and the *POPI Act* was adhered to regarding obtaining personal information of participants. Permission for the utilisation of the venue, such as an office at the healthcare institution, for the interviews was obtained from the relevant manager by the authors. Mediators such as psychologists, occupational therapists and speech therapists assisted with the recruitment process when participants brought their children for appointments in the facilities. The mediators provided information to the participants about the research study. The mediators also introduced, liaised and facilitated communication between the first author, the independent person and the participants. Those who were interested in participating informed the mediators and the mediators informed the independent person and the first author. The first author used the services of an independent person who was not involved in a study and provided the necessary training. Training included how to explain and obtain informed consent from the participants. The independent person provided the participants with the consent forms, explained their rights regarding participation in the study, and provided them with the opportunity to read the consent form at home. Participants were informed verbally by the independent person that the consent is voluntarily, and that those who did not want to participate would not be penalised. All participants participated voluntarily in the study, with no coercion. Participants were provided with the opportunity to ask questions for clarification. The independent person obtained signed consent and the mediator acted as a witness 48 h after their decision to participate in the study. The consent stated the nature and the purpose of the study, objectives and expectations, consequences, risks and benefits involved (including mode of data collection, which was individual face-to-face semi-structured interviews) and consent by the participant to be audio-recorded. The contact details of the first author were provided to the participants in case of questions or clarifications. Participants were informed that the final transcribed data would be archived at the North-West University (Potchefstroom) to maintain confidentiality. Hard copies, such as signed consent forms, would be stored in locked cupboards for a period of 5 years in the director’s office of the research entity and were not to be used for future research. The independent person then submitted the consent forms to the first author who conducted the interviews. The study was initially self-funded, then later bursary-funded after data collection. No direct gains, remunerations or incentives were provided. Participants’ submissions were summarised and ratified through member checking for confirmation before the interview ended.

### Data analysis

The recorded data were transcribed verbatim. The interviews that were conducted in South Sotho were transcribed verbatim and translated into English using translation applications by the first author and were then translated back to South Sotho to confirm that the data resembled the original. The accuracy of the transcribed data was ensured by the first and second authors by listening to the recordings and cross-checking with the transcripts.

Data were analysed using a reflexive thematic analysis method, which enabled the first and second authors to search for themes that emerged from the transcriptions (Ward, Comer & Stone 2018:142). The method is based on six steps of Braun and Clarke’s thematic analysis: (1) familiarisation, (2) coding, (3) generating initial themes, (4) reviewing and developing themes, (5) refining, defining, and naming themes, and (6) writing up (Braun & Clarke 2020:39).

The audio-recorded data were transferred to an encrypted and password-protected computer file for confidentiality and privacy and was deleted from the devices after transcriptions. The second author and co-coder completed and signed the confidentiality agreement letters prior to handing over the transcribed data. The co-coder assisted with coding and analysis of data. Meetings were held separately for both the first author and the co-coder to confirm the analysed data.

### Trustworthiness

To ensure the replication of data in the research study using the same participants, coders, and settings, it is necessary to include a detailed description of the study, establish an audit trail, and replicate the study in a stepwise manner (Forero et al. 2018:3). The first author ensured dependability by conducting a repeatable research process by implementing in detail the study process and methods that could make it possible for the study to be repeated.

Confirmability was achieved through participants’ discussions and transcripts for independent analysis. Transferability in qualitative research means findings can be applied to different contexts or populations (Daniel 2019:4). The results of the study may be transferred to other settings to see if they provide the same results. The first author described in detail the study’s context, sample characteristics, participants’ experiences, recruitment methods and data types. The correctness of the qualitative self study was also improved by means of member checking, which gives the researcher a tool to confirm the genuine meaning of participants’ voices (Candela 2019:620). The participant’s conversation and discussion were reiterated and summarised before the interview ended to verify the information discussed.

Self-description and self-reflection were used by means of a reflective journal to reduce bias and acknowledge the researcher’s thoughts, ideas, and beliefs (Shufutinsky 2020:54). The first author acknowledged the potential for bias because of her being a parent to a child diagnosed with ASD.

### Ethical considerations

The Scientific Research Committee of North-West University authorised the scientific approval to conduct the research through the Health Research Ethics Committee (NWU 0002622A1). The provincial Department of Health authorised the authors to obtain goodwill permission from the clinical managers to conduct the study. An online training programme on Ethics (Training and Resources in Research Ethics Evaluation), on how to conduct research responsibly and ethically, was completed. Confidentiality was adhered to by ensuring that the participants’ personal information was protected. To ensure anonymity, each participant was allocated a number (Participant 1 or 2, etc.), and their identity was not divulged during the discussion of findings. Every research member involved in this study completed confidentiality agreement forms. The principle of beneficence, according to Valizadeh et al. (2022:3) is mentioned as doing good, kindness and charity or any action that benefit others. Participants were treated with utmost respect throughout the research process. The term ‘non-maleficence’ aims to minimise risk and prevent harm to society and study participants (Pietilä et al. 2020:5). Participants were provided with information on potential risks associated with the study, including stress and depression, as well as the availability of psychological help in the event of a need. According to Motloba (2018:418), autonomy is the freedom to make decisions independently, without external influence, and it comes with various rights and privileges. Participants were provided with thorough information (the nature and effect of the study, the objectives and the expectations, consequences, the risk, and the benefits) and with opportunity to decide to participate or not without any coercion. According to Pietilä et al. (2020:53), principle of justice entails that participants should be treated equally and fairly. Participants were selected according to the selection criteria and given an equal opportunity to take part in the research study. There was no discrimination against any participant based on gender, age or ethnicity. The research study did not include the participants listed in the exclusion criteria.

## Results

Socio-demographic factors such as age, marital status, age of the child with autism, employment status, educational level and support structure were collected, as these characteristics are believed to play a crucial role in how caregivers respond to their experiences, and the coping mechanisms they adopt. This followed the conclusions of a study by Wang et al. (2022:3) that the above-mentioned characteristics have some impact on experiences and coping mechanisms of caregivers – and they were therefore included in the study.

A total number of ten (nine females and one male) caregivers participated in the study. Participants were Africans, aged 28–50 years, six were unemployed and four employed, four participants were married, two divorced and four single. The educational levels ranged from high school to tertiary levels and the children’s ages ranged between 3 and 17 years. Participants were caregivers who usually brought their children for intervention through occupational, psychological and speech therapies at the hospital.

Data analysis yielded two themes with sub-themes as illustrated in Table 2, which are subsequently discussed, strengthened by participants’ verbatim quotes and evidence from the literature.

### Theme 1: Caregiver’s experiences in raising a child with autism

Caregivers reported various experiences in raising a child with ASD. The experiences yielded six subthemes: (1) Understanding the child’s social and behavioural communication, (2) increased responsibility decreased self-care and freedom, (3) emotional taxing, (4) social discrimination and stigma, (5) financial implications, and (6) access to educational schools.

#### Subtheme 1.1: Understanding behavioural and social communication

Participants mentioned difficulty in understanding the child’s behavioural and social communication. They also stated that a child with autism behaves differently than a normal child because of significant struggles in communicating their needs. Children with autism exhibit impairments in behavioural and social interactions. They may communicate through certain behaviours such as aggression, tantrums, sign language and pointing at objects to make their needs known. These micro-aggressions generate parental stress, especially if there is misunderstanding in communication. On occasion, caregivers reported that the child could become aggressive and destructive, complicating the capacity of caregivers to understand the meanings of these outburst behaviours. Caregivers confirmed these socio-behavioural challenges through the following vignettes:

‘I must explain everything, that [*child’s name*] behaves like this, it’s because of certain things …’ (P1, Female, Married)

‘… Some say that he is aggressive, naughty, and destructive. Others take him the way he is …’ (P5, Female, Single)

‘… Somehow, he harms himself in the house, when he is angry, he can take something, he can throw himself on the ground, or he can stab himself, he injures himself, it is like you should watch him for … signs like that, watch out for signs like that, even if … make sure that the match sticks are placed away.’ (P7, Female, Single)

Children with autism are more inclined to exhibit challenging behaviours than children with learning disabilities, intellectual disabilities or typically developing children (Ang & Loh 2019:2129). According to Alli et al. (2015:81), developmental changes, communication difficulties and behavioural patterns that occur in children with ASD cumulatively affect parental and family functioning, resulting in elevated stress. The caregivers confirmed these communication challenges through the following vignettes:

‘I have experienced many things because this child did not know how to speak. She will just cry, sometimes, when she needs water, she will come and point at the water, sometimes she just points, then I will know my child is thirsty. How my child behaves when she is hungry? when she is sick, I should know what to give, and what to do to her.’ (P2, Female, Divorcee)

‘So, you must have your way of communicating with him to understand each other …’ (P7, Female, Single)

‘When he was between 2 and 3 years old, because he didn’t speak … it was difficult for me because I didn’t understand, and his development was slow, I did not understand that by his age he couldn’t speak clearly what is happening.’ (P9, Female, Divorced)

#### Subtheme 1.2: Increased responsibility decreased self-care and freedom

Caregivers indicated that parenting a child with autism comes great responsibility and pressure; hence, caregivers do not have the freedom and time for their own health and general wellbeing as they must provide continuous close supervision of the child:

‘It’s a problem, because everything I plan, I always put her first. Whenever I need to go somewhere, I should find who can babysit her for me.’ (P6, Female, Married)

‘You [*the caregiver*] experience that time of wanting freedom, so most of the time it’s pressure, whereby, you always have, wherever you are, there is the child.’ (P7, Female, Single)

‘… If, you [*the caregiver*] don’t take care of yourself, mentally, physically, emotionally, I realised that it will be difficult for me to carry this young man, who is having this condition.’ (P9, Female, Divorced)

The findings reported by Aguiar and Pondé (2019:46) confirm that the majority of parents, especially the mothers, experienced emotional and physical overload that complicated the processes of caring for their general well-being and their health in particular. This is also verified in the findings of McAuliffe et al. (2019:7) who observed that parents of disabled children failed to consider personal requirements, such as health issues, because of a perceived lack of time.

#### Subtheme 1.3: Emotional taxing

Participants in this study reported feelings of helplessness and depression, and being stressed, exhausted and emotionally drained because of failure in managing child’s behaviour. The following direct statements support this observation:

‘It’s very, it’s very hard. His mother is on depression medication.’ (P3, Male, Married)

‘I feel that I don’t know what to do anymore … true.’ (P6, Female, Married).

‘Sometimes they give me like, exhaustion, I’m tired, like I get tired, I become stressed sometimes … I fail to have patience.’ (P7, Female, Single)

‘It’s like he has a lot of anger issues, so eish, at times, as a parent, it affects me a lot, like, they tend to hurt me, sometimes I ask myself, “why me”? It is emotionally draining.’ (P8, Female, Single)

The findings of Nik Adib et al. (2019a:7) confirm that caregivers of children with ASD are more likely to have high stress levels when compared with caregivers of children with other disabilities. These findings are in accordance with those of Salomone et al. (2018:1) that compared to parents of children with other developmental or physical disabilities, parents of children with autism report higher rates of stress and mental health problems. This could also pertain to the significance of parental involvement in managing a child’s challenging behaviors, leading to stress, fatigue, and emotional depletion.

#### Subtheme 1.4: Social discrimination and stigma

Society does not understand the causes of the child’s challenging and difficult behaviours; as a result, ASD children are not treated the same way as other children. Caregivers experienced stigmatisation, such as the child being labelled mad, and social rejection and isolation which negatively affected them. Caregivers reported a lack of support structure in their experiences. This was affirmed by participants through the following quotes:

‘Other members of the family do not treat him like other children, they regard him as a mad child, you, see.’ (P7, Female, Single)

‘Umm, and the society, the society is very cruel when they do not understand something. Like the neighbours, a person will not be understanding what causes the child’s behaviour … And the challenges I had, that is, I wanted to isolate him because people do not understand him.’ (P10, Female, Single)

‘… [*P*]eople don’t know, people usually concentrate on the child, forgetting that you as a mom, and you need a support too … there needs to be a support structure amongst us as parents of autistic children.’ (P9, Female, Divorced)

A lack of support structure from the families and society resulted not only in social isolation, guilt and shame of having a child with ASD (Lamba et al. 2022:2) but also in isolation and stigmatisation (Cloete & Obaigwa 2019:4). People’s reactions such as shock, anger and blame of the inappropriate behaviours of children with ASD cause parents to feel shame and embarrassment (Zhou, Wang & Yi 2018:1). Shame and embarrassment consequently result in social isolation of parents themselves.

#### Subtheme 1.5: Financial implications

Caregivers described financial constraints because of the expenses incurred by the child, such as expensive private school fees, transport and food. Caring for a child with autism has a negative impact on caregivers as indicated in Table 1, as six out of ten participants reported being unemployed, and the child being expensive to maintain financially. This strains financial resources, making it difficult to raise a child with autism. Shattnawi et al. (2020:6) also assert that most mothers described financial stress emanating from having a child with ASD. Financial stress essentially means that parents with such children require substantial assistance with finances and resources:

‘The child with autism, I want to tell you, they are expensive. Sometimes, she can just be sick out of nowhere. The private schools, they are expensive.’ (P2, Female, Divorcee)

‘She started receiving the grant money. The grant money pays for the fees, and transport and again she uses Pampers, she is expensive.’ (P6, Female, Married)

‘Eish! finances are a bit difficult because the lifestyle he lives is not the one I live, He is a picky eater, He’s got things that he likes. And as a parent, you will also want your child to have all the things he wants, as much as sometimes I deny giving him.’ (P9, Female, Divorced)

‘Yes, but I am also looking for a job so I could work too.’ (P4, Female, Married)

**TABLE 1:** Sociodemographic qualities of participants.

Participant number	Gender	Age (years)	Marital status	Educational level	Employment status	Participants’ support structure	Number of children	Child’s age (years)	Child’s gender
P1	F	47	Married	Tertiary level	Employed	Sister and mother	4	11	M
P2	F	50	Divorcee	Tertiary level	Employed	Mother	3	11	F
P3	M	50	Married	High School	Unemployed	Wife and children	4	6	M
P4	F	34	Married	High School	Unemployed	Husband	4	4	M
P5	F	37	Single	High School	Unemployed	Mother	5	6	M
P6	F	35	Married	Tertiary level	Unemployed	Husband and mother	2	4	F
P7	F	34	Single	High School	Unemployed	Partner	2	7	M
P8	F	28	Single	High School	Unemployed	Creche	1	6	M
P9	F	42	Divorced	High School	Employed	Parents, son, younger sister, daughter, child maintenance	2	10	M
P10	F	42	Single	Tertiary level	Employed	Mother	4	16	M

*Source:* Ang, K.Q.P. & Loh, P.R., 2019, ‘Mental health and coping in parents of children with autism spectrum disorder (ASD) in Singapore: An examination of gender role in caring’, *Journal of Autism and Developmental Disorders* 49(5), p, 2132, https://doi.org/10.1007/s10803-019-03900-w

F, female; M, male.

**TABLE 2:** Themes and subthemes.

Themes	Subthemes
1. Caregiver’s experiences in raising a child with autism	1.1. Understanding behavioural and social communication 1.2. Increased responsibility decreased self-care and freedom 1.3. Emotional taxing 1.4. Social discrimination and stigma 1.5. Financial Implications 1.6. Access to educational schools
2. Caregivers’ coping in raising a child with autism	2.1. Understanding the child’s communication patterns 2.2. Support from family members 2.3. Emotional coping as a caregiver 2.4. Isolation as a negative coping 2.5. Healthcare support 2.6. Financial resources

#### Subtheme 1.6: Access to educational schools

Participants reported difficulty in finding appropriate schools as schools are constantly in distant locations. The fees are high, while waiting lists for admission are an encumbrance. This is complicated by assessments to establish neurodevelopmental conditions that must be conducted if the child should be assisted into mainstream schools. The following quotations affirm the encounters caregivers faced:

‘There are no schools, my child goes from here to far away, and the next thing, they’ll be telling me that, it’s weekend out, I should go fetch her, rather if she was close and [*I would*] be able to know what is happening.’ (P2, Female, Divorcee)

‘I wish they can open a public schools because, I know most of the autism schools are private. Some of us can’t afford them.’ (P6, Female, Married)

‘Even with their schools, to find a school for a child you must be on the waiting list. They assess the child, and after assessing him again it’s another waiting list so that they can create a placement for him in the school, so it takes some time you see.’ (P7, Female, Single)

‘… At the government school, they rejected him. They say that he is a difficult child, and it is not their responsibility to handle an autistic child.’ (P8, Female, Single)

A lack of educational facilities strains caregivers financially, as they must identify schools in other areas, which further increases expenses such as transportation. According to caregivers in this study, the schools that are currently available are expensive and private (i.e., not government-run). Assessments and waiting lists are frustrating as it takes time to complete the process, and there is no guarantee that the child will be accepted at the school. There is rejection of children with ASD in government schools because of difficult behaviours and teachers’ inability to handle an autistic child. Lamba et al. (2022:8) indicated difficulty in finding educational and healthcare facilities because of a rise in the prevalence of ASD, and consequently caregivers are compelled to shuttle between hospitals, clinics and educational institutions, which further complicate their financial and psychological burdens.

### Theme 2: Caregivers’ coping in raising a child with autism

According Walke, Chandrasekaran and Mayya (2018:2), coping is defined as ‘constantly changing cognitive and behavioural efforts to manage the specific external or internal demands that are appraised as taxing or exceeding the resources of the person’. The following subthemes were identified:

#### Subtheme 2.1: Understanding the child’s communication patterns

According to Doak’s (2021:207) findings, parents could identify in their children facial expression, vocalisation, object manipulation, proxemics (body language), haptics (touch), and posture. Although there are challenges in coping, parents have learned through experience to recognise the child’s way of communicating basic needs. Caregivers had to learn their children’s individualised communication patterns, understand gestures, language and expressions so that they could overcome communication barriers (Doak 2021:207). The following quotes confirm their frustrations:

‘She will just cry, sometimes, when she needs water, she will come and point at the water, sometimes she just points, then I will know my child is thirsty.’ (P2, Female, Divorcee)

‘So, you must have your way of communicating with him to understand each other. He should know that mommy, okay, if I do something wrong, mommy doesn’t want me to do that. Yes, if I do that, she will scold me.’ (P7, Female, Single)

‘… [*h*]e will be going to take the bowl from the cupboard and put it on the table. I will be knowing that he is hungry.’ (P8, Female, Single)

The behavioural and social communication deficits are particularly stressful for parents of children with autism (Ang & Loh 2019:2130).

#### Subtheme 2.2: Support from family members

As the responsibilities and pressures of caring for children with ASD increases, some caregivers receive support from family members such as a mother or other members, for example, a twin brother, while the caregiver is attending to errands, which provides a sense of relief. Some of the caregivers reported not receiving any support from family members, which resulted in chronic sense of loneliness:

‘Mine has a twin brother; the twin brother helps me.’ (P1, Female, Married)

‘I leave her with my mother when there are errands. She is the one who supports me the most.’ (P6, Female, Married)

‘Sometimes I feel so alone as if family is not helping me.’ (P8, Female, Single)

‘My sibling and my relatives, they are very supportive.’ (P10, Female, Single)

Family support positively impacts the social and psychological well-being of caregivers by reducing stress and work overload of parenting a child with autism.

#### Subtheme 2.3: Emotional coping as a caregiver

Emotional coping of caregivers included receiving emotional support from friends and application of self-relaxation techniques. This is confirmed by the following vignettes:

‘Friends assist me. I have friends that have autistic children. So that friend of mine has been supporting me a lot emotionally.’ (P1, Female, Married)

‘I don’t do anything, I sit on the floor and then drink water, I just sit on the floor, until I feel okay, and my mind relaxes little by little.’ (P7, Female, Single)

Selvarkumar and Panicker’s (2020:230) findings indicated that mothers adopted active coping, positive reframing, planning, acceptance and religious-practice as their primary coping mechanisms.

#### Subtheme 2.4: Isolation as a negative coping

Participants reported having to use isolation as a negative coping mechanism by means of self-isolation or isolating the child, to avoid being around people and to have to explain the child’s conditions. People were also isolating themselves from the participants. The following vignettes affirm isolation as a negative coping mechanism applied by caregivers:

‘… immediately you enter the church, they isolate themselves from you. We are isolated from people.’ (P2, Female, Divorcee)

‘It was difficult for me to explain to everyone, the condition of my child, so I preferred to lock him inside because some of the things I did not understand.’ (P10, Female, Single)

‘I had to isolate myself socially and avoid being around people.’ (P9, Female, Divorced)

Social support may reduce the stress related to raising a child with ASD as it closes the gaps created when formal services withdraw from emotional and practical support (Pepperell et al. 2016:7). Caregivers’ involvements in social interactions provides emotional support and self-esteem, which are important for adjustment and resilience (Hawken, Turner-Cobb & Barnett 2018:5).

#### Subtheme 2.5: Healthcare support

Caregivers reported receiving healthcare support from healthcare practitioners such as psychologists or speech and occupational therapists. This was supported by the following quotes:

‘You take her for speech therapy and take her for O.T. [*occupational therapy*].’ (P2, Female, Divorcee)

‘I take the child to a speech therapist, occupational therapist.’ (P7, Female, Single)

‘Mostly I have been helped a lot by psychology.’ (P8, Female, Single)

Access to healthcare and support for children with ASD is still inadequate globally, caregivers rely on healthcare professionals, especially doctors, to provide information and to discuss problems concerning their ASD child (Nik Adib et al. 2019b:13). The expert support can assist and empower in the communication aspects to improve caregivers’ understanding of how best to assist their child’s communication (Laubscher 2022:88).

#### Subtheme 2.6: Financial resources

Caregivers in this study indicated that they were consistently in financial difficulties. They reported that they had to either save money or withdraw from their paltry savings. Most caregivers acknowledged receiving financial assistance from the government as some form of social relief, which assisted with their financial needs. Some of the caregivers were employed but most were unemployed, which added more strain on their finances:

‘I have to save money, since … I am the one staying with the children. So, I use money from my savings, so that he can be like other children and have fun.’ (P1, Female, Married)

‘She started receiving the grant money. The grant money pays for the fees, and transport and again she uses Pampers, she is expensive.’ (P6, Female, Married)

‘At least money from SASSA, it is at least [some stipend] that can cover most of the things and it meets me halfway for helping with my child.’ (P8, Female, Single)

‘And very expensive and some of the things he wants, they are not even around here, we must drive, it’s petrol for that, but you know, I know, maybe God is great. There is school fees, there is transport, the … He grows up every day, like, the shoes, there is uniform … The clothes to buy, food and then there’s … [*a nanny*] to pay … because I am a working mom.’ (P9, Female, Divorced)

Caregivers reported receiving money from SASSA (South African Social Security Agency), which is a national agency that provides social assistance as income transfer in the form of grants (Vally 2016:965). The South African government established social grants to increase the financial security of poor households, to reduce poverty and increase access to food (Chakona & Shackleton 2019:2). The social grant assisted caregivers in meeting some of their financial needs, despite the child being expensive, financial needs can be in terms of school fees, transport, shoes, uniform, clothes, food and paying of the child’s nanny. This report is consistent with the findings of Erasmus, Kritzinger and Van der Linde (2019:13) who confirmed that families parenting children with ASD experienced substantial economic strain and required additional funding from the government.

## Discussion

The study yielded two themes that answered the research questions: What are the experiences of caregivers of children with ASD in North West province, South Africa? How do caregivers of children diagnosed with ASD cope personally, emotionally, socially and financially? The study is derived from the 1992 conceptual framework of the socio-ecological model by Bronfenbrenner. The discussion of the study is based on the five levels of the framework.

The first level of the framework focuses on the individual characteristics such as age, gender, education, economic resources, expectations and values, which influence caregivers’ responses to experiences and coping mechanisms. Caregivers of children with ASD reported experiences that impact different aspects of their lives including emotional, social and financial aspects, as well as challenges in finding appropriate educational facilities for their children as expectations. The study of Lamba et al. (2022:2) stated that social, physical, psychological and financial challenges experienced by parents of children with ASD lead to an increase in parental stress, poorer family functioning, and higher levels of conflict compared to parents of typically developing children or parents of children with other developmental disabilities. Caregivers reported that a child with ASD often exhibits social and behavioural communications that are different from a normal child and are difficult to understand, which resulted in feelings of helplessness or depression, and being stressed, exhausted and emotionally drained. Similar to our study’s findings, Ilias et al. (2016:75) indicated that caregivers reported higher levels of stress and depression as a result of difficulty in dealing with their child’s behaviour. Furthermore, these findings are consistent with Prevedini et al. (2020:3) who submit that caring for a child with neurodevelopmental disorders or any other disability exposes parents to a greater physiological and psychological response to stress, particularly if the child exhibits inappropriate behaviour or socio-communicative impairments. Caring for a child with autism comes with increased responsibility and intense pressure, leaving caregivers with less time and freedom to focus on their own health and general well-being. Caregivers indicated the need to have individual time and the importance of taking care of their mental, physical and emotional well-being, to care for the child successfully. The aforementioned is consistent with the results of Aguiar and Pondé (2019:5) who verified that the majority of parents, particularly mothers, experience severe emotional and physical overburdening that affects their capacity to take care of themselves and their health in general. Other studies have also indicated that mothers of children with autism experience higher levels of stress than fathers (Burrell et al. 2017:1135; Salas et al. 2017:59).

The second level in Bronfenbrenner’s framework is based on interpersonal relations, which includes formal and informal social networks and support systems such as family, friends, social support and healthcare professionals, who play an important role in supporting caregivers and promoting positive well-being. The findings of this study indicated a mixture of positive and negative family support, as some caregivers were receiving support from family members and others felt neglected. These findings are consistent with the findings of Shorey et al. (2020:6) who also arrived at the conclusion that many parents experienced rejection and criticism from extended family members for having a child with autism. Other caregivers felt adequately supported by their partners and extended family, who helped to care for their child (Shorey et al. 2020:7).

The findings indicated caregivers of children with ASD experiencing stigmatisation, as the child is labelled insane and is rejected by some family and society members, resulting in social isolation. As in the study’s findings of Tushar et al. (2020:5) members of immediate and extended family, close and distant relatives or acquaintances frequently displayed harsh, stigmatising attitudes towards persons with autism, which negatively impacts on persons with autism, family members and especially mothers. Furthermore, mothers of children with autism avoid social events because of discrimination or embarrassment (Aguiar & Pondé 2019:46). Caregivers received healthcare support from services such as speech therapy, occupational therapy and psychology for their children. Caregivers in this study relied on various sources of support, including family members, friends and healthcare services such as psychologists and occupational therapists, in addition to employing social isolation as coping strategy. The aforementioned are consistent with the findings of Pepperell et al. (2016:7) where mothers depended more on the support of others, such as family, friends and professionals. Caregivers emphasised the need for the support structure.

The following phase entails engaging with the community. Caregivers affirm that there is a deficiency in societal knowledge and awareness concerning autism, accompanied by a lack of comprehension regarding the obstacles encountered by caregivers. Generally, the focus is directed towards the child.

The institutional level is the fourth level, in which caregivers expressed that raising a child with autism is difficult and expensive because of unemployment and additional expenses such as school fees and uniform, transport, clothes and food. This is consistent with the findings of Samadi and Samadi (2020:3) who confirmed that unemployment has been added to the list of new risks to overall well-being and the level of stress experienced by caregivers of people with ASD. Other study’s findings indicated that children with ASD use healthcare services more frequently than children without ASD, and their parents are financially more burdened by healthcare costs than parents of children with other special healthcare requirements (Wilson & Peterson 2018:11). Although it has been demonstrated in many instances that having a child with autism increases parental stress, this association is incomplete because other stressors unrelated to having a child with autism are not considered (Bluth et al. 2013:199).

Further to the discussion of the institutional level, caregivers reported a lack of affordable educational facilities specifically for children with autism, which is consistent with the findings of Shorey et al. (2020:7) where parents complained of the lack of a comprehensive educational system. Caregivers reported that available facilities are either expensive or not accessible and mainstream schools reject their children because of inappropriate behaviours and inadequate training of educators to manage children with autism. It is also a long process to get a child with autism to be accepted in special schools because of assessments and waiting lists. Furrukh, Anjum and Navalta (2020:13) stated that some mothers had negative experiences with assessments and therapists, and subsequently had to go through a lengthy process to find appropriate schools that would accommodate their children’s specific needs.

The fifth level is the policy/enabling environment, which in this study indicates that caregivers of children with ASD receive minimal financial assistance in the form of grants to care and support for their children. The SASSA provides social support as income transfer in the form of grants (Vally 2016:965). Social grants increase the financial security of poor households, reducing poverty and increasing access to food (Chakona & Shackleton 2019:2). Social grants support provides minimal financial assistance that enables caregivers to provide basic needs for their children.

### Limitations

The study had only one male participant; therefore, caregiver’s experiences and coping mechanisms are based on the mothers/females experiences rather than fathers/males who might have provided more data from the male perspective – this is a consequence of being in a society where childcare is typically more the responsibility of mothers rather than fathers. Although mothers are considered the primary caregivers in most cultures, different caregivers such as aunties, grandparents and uncles in the family might have different experiences in the process of caring for an individual with ASD at home (Samadi & Samadi 2020:7). Only one father and nine mothers participated in the study. Recruitment from wider geographical area may help future studies to sample the diverse caregiver experiences.

Depending on the severity, functional status and presence of coexisting conditions, the needs of people with ASD are complex and varied (Marsack-Topolewski & Graves 2019:72). As ASD is a spectrum, each presentation of ASD varies vastly from one child to another. Children with autism present differently depending on the age and the level of the autism severity (our study inclusion criteria ages were from 3 to 17 years, and the functional state was excluded), which may provide different study findings. The accuracy of the findings for other caregivers in different contexts cannot be presumed, although the research process has been sufficiently outlined to enable replication of the study.

Recommendations include the establishment of support groups to assist caregivers in sharing ideas regarding communication strategies and management of challenging behaviours in children with autism. Support groups can provide opportunities for networking and development of support relationships among caregivers. Effective training programmes for caregiver empowerment with necessary strategies and skills to interact with their children, to manage stress and to receive counselling and support from the health care professionals are also recommended.

Further research could be conducted on topics related to caregivers and ASD, as well as the effectiveness of interventions that support the mental health of caregivers of children with ASD.

## Conclusion

The study’s objective was to explore and describe the experiences and coping mechanisms of caregivers of children with ASD in North West province in South Africa. The findings of this suggest that ASD affects various aspects of family life, including emotional, social and financial aspects, which results in feelings of stress, exhaustion and helplessness. Ultimately, caregivers of children with ASD require maximum support from family members and society, to reduce the burden of care, social isolation and stigmatisation to effectively cope in caring for their children. Support from various governmental sectors – in terms of the provision of effective healthcare services, affordable and accessible educational facilities, financial support and establishment of support groups and awareness campaigns should be prioritised.
